# Improved long-term care provision in the context of population ageing 

**DOI:** 10.2471/BLT.26.295680

**Published:** 2026-02-01

**Authors:** Jagadish K Chhetri, Barbara Kamholz, Piu Chan, Hyobum Jang, Ritu Sadana

**Affiliations:** a National Clinical Research Centre for Geriatric Diseases, Xuanwu Hospital of Capital Medical University, No. 45 Changchun Street, Xicheng District, Beijing 100053, China.; b Department of Psychiatry, Geriatric Psychiatry, University of San Francisco, California, United States of America.; c Department of Sexual, Reproductive, Maternal, Child and Adolescent Health and Ageing, World Health Organization, Geneva, Switzerland.

Population ageing is one of the major global transformations of the twenty-first century. By 2050, more than 2 billion people will be aged 60 years or older, with three quarters of this increase occurring in low- and middle-income countries.^1^ While this demographic shift reflects gains in life expectancy, a widening gap between life expectancy and healthy life expectancy means people spend later years with dependency, disability and poor quality of life, often requiring complex and sustained care.^1^^,^^2^ This gap presents challenges for health and social systems, highlighting the need for robust long-term care to support functional ability, dignity and well-being across the life course.

Despite rising care needs, long-term care remains underdeveloped and underprioritized in most countries. Strengthening long-term care is therefore a global priority, as emphasized through the United Nations Decade of Healthy Ageing (2021–2030),^3^ which calls for equitable, resilient and rights-based care for older people.

Long-term care comprises a continuum of services for people with ongoing care needs,^4^^,^^5^ including care provided at home, in communities and in institutional environments, and encompasses a mix of preventive, rehabilitative and supportive services. The World Health Organization’s (WHO) *Framework for an integrated continuum of long-term care*^5^ urges countries to embed these services within their integrated health and social protection systems to ensure continuity of care across settings.

However, long-term care systems worldwide face persistent challenges. Traditional family-based care is declining due to demographic and social changes such as smaller household sizes, labour migration and urbanization, particularly in low- and middle-income countries.^6^^,^^7^ Across all socioeconomic settings, unpaid caregivers (predominantly women) provide most care.^7^ Informal care remains indispensable but cannot replace comprehensive, high-quality long-term care systems.

Many high-income countries have formal long-term care services, but face workforce shortages, variable quality and inequitable access.^8^^,^^9^ In low- and middle-income countries, systems are often underdeveloped or absent outside major urban areas. Private services are limited, costly and insufficiently regulated, while public facilities are frequently unable to meet the growing demand. Long-term care is about care delivery and human rights, social inclusion and economic security. Weak systems expose older people to isolation, neglect or abuse, and place unsustainable financial burdens on families and caregivers.

Conversely, well-designed long-term care systems can reduce hospitalizations, strengthen community engagement, support labour force participation (especially among women), and advance universal health coverage (UHC) goals. WHO’s *Long-term care for older people: package for universal health coverage*^4^ calls for embedding these services within UHC to ensure access without financial hardship. The package provides guidance on essential interventions across care settings alongside system enablers such as governance, financing, workforce development, quality assurance, information systems and multisectoral coordination.

Several countries are advancing policy responses. Japan and the Republic of Korea pioneered social insurance models that extend long-term care through home, community and residential services, with caregiver allowances.^9^ Germany’s insurance provides cash benefits and services, alongside regulatory frameworks for quality and workforce standards.^9^ Thailand’s community-based care network is linked to primary health care, integrating preventive and rehabilitative services.^10^ China is expanding pilots that integrate medical and social care for older adults, including home-based and community-centred models.^11^ Nepal, although lacking a formal system, uses national health insurance schemes to expand service coverage for older adults in public facilities and gradually in private sectors, reducing the financial burden.^12^

These approaches demonstrate that no single model for long-term care exists, but common principles include person-centred care, system integration across health and social care, financial protection and support for caregivers. While WHO is collaborating with Member States to develop global standards, including core principles, standards for home- and community-based care, long-term care facilities and unpaid carers, as well as system enablers, the real work is in national adoption, adaptation and implementation. Doing so involves translating normative guidance into policy reform, budgetary commitments and inclusive governance mechanisms that involve older people and caregivers in decision-making.

Strengthening long-term care requires innovation, especially in low-resource settings. Community health workers can provide basic care support and link families to social services. Digital health tools can support care coordination and remote monitoring. Public–private partnerships may expand service availability while safeguarding equity and quality. Social protection schemes should align with long-term care through pension credits, caregiver allowances and subsidized services for low-income older adults.

Investing in long-term care is an investment in social resilience, health equity and human dignity. The UN Decade of Healthy Ageing provides both a mandate and momentum; decisive leadership and sustained commitment are now required to build accessible, accountable long-term care systems that meet the diverse needs of ageing populations worldwide.
